# drVM: a new tool for efficient genome assembly of known eukaryotic viruses from metagenomes

**DOI:** 10.1093/gigascience/gix003

**Published:** 2017-01-20

**Authors:** Hsin-Hung Lin, Yu-Chieh Liao

**Affiliations:** Institute of Population Health Sciences, National Health Research Institutes, No. 35, Keyan Road, Zhunan Town, Miaoli County, 35053, Taiwan

**Keywords:** next-generation sequencing (NGS), bioinformatics, metagenomics, detect, reconstruct

## Abstract

**Background:** Virus discovery using high-throughput next-generation sequencing has become more commonplace. However, although analysis of deep next-generation sequencing data allows us to identity potential pathogens, the entire analytical procedure requires competency in the bioinformatics domain, which includes implementing proper software packages and preparing prerequisite databases. Simple and user-friendly bioinformatics pipelines are urgently required to obtain complete viral genome sequences from metagenomic data.

**Results:** This manuscript presents a pipeline, drVM (detect and reconstruct known viral genomes from metagenomes), for rapid viral read identification, genus-level read partition, read normalization, *de novo* assembly, sequence annotation, and coverage profiling. The first two procedures and sequence annotation rely on known viral genomes as a reference database. drVM was validated via the analysis of over 300 sequencing runs generated by Illumina and Ion Torrent platforms to provide complete viral genome assemblies for a variety of virus types including DNA viruses, RNA viruses, and retroviruses. drVM is available for free download at: https://sourceforge.net/projects/sb2nhri/files/drVM/ and is also assembled as a Docker container, an Amazon machine image, and a virtual machine to facilitate seamless deployment.

**Conclusions:** drVM was compared with other viral detection tools to demonstrate its merits in terms of viral genome completeness and reduced computation time. This substantiates the platform's potential to produce prompt and accurate viral genome sequences from clinical samples.

## Background

Viruses are the most abundant biological entities on Earth and are found among all cellular forms of life including animals, plants, bacteria, and fungi. More than 4500 viral species have been discovered; their sequence information has been collected by researchers [1–3]. Viruses have caused some of the most dramatic and deadly disease pandemics in human history and viral disease outbreaks tend to occur every several years. Over the past two decades, avian influenza H5N1 virus [4], SARS coronavirus, H1N1 pandemic, MERS coronavirus [5], Ebola virus [6], and Zika virus [7] have emerged in the human population. During such outbreaks, identification of the causative agent and comparative genome analysis is of cornerstone importance for disease surveillance and epidemiology. Unbiased next-generation sequencing (NGS) is emerging as an attractive approach for viral identification from varied samples, including blood, feces, sputum, and other swab samples [8, 9]. The technique holds the promise to aid in the identification of potential pathogens in a single assay without a prior knowledge of the target [10]. However, computational analyses for crude metagenomic deep-sequencing reads are extremely time-consuming.

SURPI [10] and Taxonomer [11] are pathogen detection tools that have been proposed to rapidly analyze metagenomic NGS data for comprehensive diagnostic applications. However, both tools are incapable of complete viral genome assembly. VIP pursues the same strategy as SURPI, subtraction to identification, to subtract host and bacteria reads prior to the identification of viral reads, although it does provide an alternative strategy to the assembly of reads under a genus, classification to assembly, hence enabling improved viral genome assembly [12]. Although VIP generates phylogenetic trees, thereby facilitating the visualization of genealogy between a candidate virus and existing reference sequences, it does not produce assembled viral sequences in its final report. Moreover, ease-of-use challenges associated with operating SURPI and VIP impede their applications in most laboratories and are often accessible only by trained personnel. VirusTAP is a web-based integrated NGS analysis tool for viral genome assembly from metagenomic reads [13]. This user-friendly tool enables users to obtain viral genomes more easily by merely uploading raw NGS reads and clicking on several selections. However, VirusTAP only accepts Illumina data and does not support users to update reference databases. Therefore, tools for viral metagenomics analyses are urgently required that are substantially more computationally efficient, accurate (for compete viral genome assembly), and easy to use.

Here we present drVM (detect and reconstruct known viral genomes from metagenomes), a bioinformatics pipeline that first provides rapid classification of NGS reads generated by Illumina or Ion Torrent sequencing technology against a viral database, then partitions the viral reads into genus groups, and finally *de novo* assembles viral genomes from the corresponding genus-level reads. For ease of deployment, a Docker container [14], an Amazon machine image, and a virtual machine [15] image were created for drVM. The performance of the platform was evaluated in the analysis of 349 sequence data in the Sequence Read Archive (SRA) from 18 independent studies [8–10, 13, 16–29]. These data sets encompass a variety of sample types, viruses, and sequencing depths. drVM was demonstrated to be highly adept at the detection and reconstruction of various known viral genomes and concomitantly outperforms other analytical pipelines including SURPI, VIP, and VirusTAP.

## Materials and Methods

### Reference databases

Viral nucleotide sequences were obtained from the Nucleotide database of the National Center for Biotechnology Information (NCBI) using the query term “(complete[title]) AND (viridae[organism])”; this query resulted in 642 079 hits (as of 16 March 2016) and increased to 705 577 hits (as of 20 October 2016). Based on each sequence identifier (accession number) of the viral sequence, its taxonomic ID, scientific name, and genus-level annotation were separately obtained from the NCBI Taxonomy database. Viral sequences with taxonomy ID not included in the virus division (division id = 9), those with taxonomic information absent, and those lacking genus-level annotation were labeled “nonViral, “noTax,” and “noGenus,” respectively. The noGenus sequences can be used by adding “-kn on” option in the creation of databases. With the exception of noGenus sequences, those sequences with the nonViral or noTax labels were excluded from database construction. The remaining viral sequences were utilized as rawDB. Three hundred and seventy-seven viral genomes were obtained by filtering with host “human” from the NCBI viral genome resource to retrieve 684 sequences (1 March 2016) [1]. The viral sequences were segmented into their corresponding genus level for refDB; the corresponding refDB was uploaded to SourceForge for later use (https://sourceforge.net/projects/sb2nhri/files/drVM/refDB.tar.gz). To increase read alignments and reduce memory requirements, rawDB was split into eight sub-databases, and each contained sequences with total length <200 Mbp. SNAP (version 0.15.4) [30], possessing a default seed size of 20, was used to generate index tables (snap_index_virus) for the viral sub-databases. In addition, a BLAST database (blast_index_virus) was created by makeblastdb (BLAST 2.2.28+) [31] from rawDB to enable contig annotation.

### Implementation of drVM

**Figure 1.**
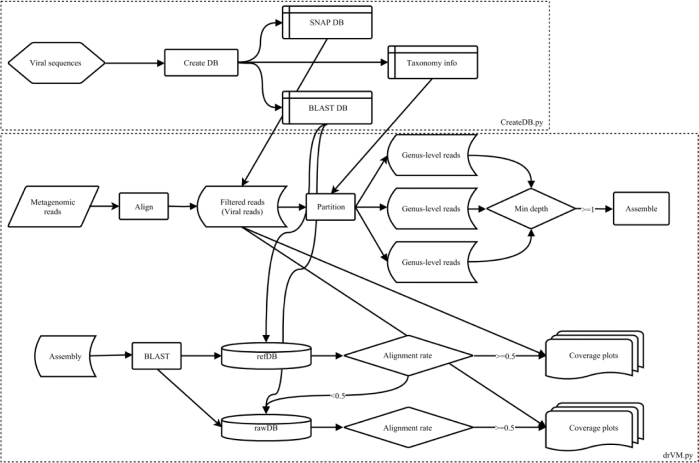
A schematic flowchart of drVM. CreateDB.py processes viral sequences to produce SNAP and BLAST databases. drVM.py analyzes NGS reads to produce viral genomes.

The drVM pipeline is implemented in Python and incorporates several open-source tools, including BLAST, SNAP, and SPAdes [32]. The package comprises two modules: CreateDB.py and drVM.py. A schematic flowchart of drVM is shown in Fig. 1. In CreateDB.py, viral sequences (provided by the user) are processed to produce rawDB for SNAP and BLAST database construction, and refDB is downloaded from the SourceForge link. drVM.py takes single- or paired-end reads as input. It aligns reads to SNAP DB and, accordingly, identifies viral reads using the following parameters: snap single -x -h 250 -d 24 -n 25 -F a. Based on taxonomic information (genus-level annotation) of the aligned sequence, the viral reads are partitioned into genus-level groups. Each group is labeled according to its corresponding genus. By examining the aligned sequences with reads, sequence coverage and read depth are calculated. Maximum coverage and average depth for a genus are estimated by taking all aligned sequences corresponding to that genus into consideration. Viral reads in a genus with average depth more than or equal to min depth (default = 1) undergo *de novo* assembly via SPAdes (v.3.6.1). Prior to assembly, digital normalization [33] in khmer [34] is employed to remove reads with high-abundance (default = 100) k-mers. Using BLAST, each genus-level assembly is annotated to its closest reference against refDB using blastn (identity ≧80%). Each reference is examined to confirm that it contains at least a 50% alignment rate. If this procedure returns no hits, the assembly is annotated by BLAST against rawDB. Note that, although the viral sequences in refDB are restricted to human viruses, there is no filter by host option in rawDB. Reads are aligned to contigs by means of SOAP2 [35] and the read-covered contigs are placed into a coverage plot with reference-guide coordinates. To increase contig continuity, reads aligned to contigs corresponding to a reference are extracted for pairs and those paired reads are re-assembled. This re-assembly process is only performed for paired-end reads.

### Simulation datasets

A reference genome of the hepatitis C virus (HCV; NC_004102.1) was used to simulate metagenomes with 10×, 15×, 20×, 30×, 40×, and 50× viral reads using VirtualNextGenSequencer [36]. The simulated reads were concatenated to 1-Gbp sequencing reads (3.5 million paired-end 150-bp reads) of liver cancer cells (SRR3031107 in SRA) and 328-Mbp reads of a human microbiome sample (SRR062412, 100-bp paired-end reads containing any Ns were removed). The reads of liver cancer cells and the reads from the human microbiome project (HMP) were taken as host and bacterial metagenomes. Three reference genomes of respiratory viruses including human enterovirus (NC_001612 for CA16), human rhinovirus (NC_001617 for HRV), and human respiratory syncytial virus (NC_001781 for HRSV) were used separately to simulate 20×, 20× and 40×, and 20× and 80× viral sequences using VirtualNextGenSequencer. The simulated reads (all 20×, and 20×-40×-80×) and randomly selected reads (1 Gbp, 5 million paired-end 100-bp reads), as a background, from an influenza-negative sample (ERR690488) [9] were used to assess the ability of drVM to assemble viral genomes within the same genus (human enterovirus and rhinovirus).

### Metagenomic datasets

A total of 349 sequencing runs in the SRA were downloaded (see Table S1). The metagenome datasets based on prior studies [8–10, 13, 16–29] are summarized in Table 1. Each run was analyzed by drVM: drVM.py -1 read1.fastq -2 read2.fastq -t 16 (-type iontorrent for Ion Torrent datasets) on a server (Intel Xeon E7-4820, 2.00GHz with 256 GB of RAM).

**Table 1.** The feasibility of drVM in various types of clinical samples and public datasets

				drVM result (No. of run)	
Study description	Sequencing method	No. of runs	Ref	Detection	Reconstruction
Bioinformatics pipeline for ultrarapid pathogen identification	Illumina	13	[10]	6	0
Complete viral RNA genome sequencing of ultra-low copy samples	Illumina	34	[19]	9	25
Faecal virome of red foxes from peri-urban areas	Illumina	8	[28]	0	1
Full genome virus detection in fecal samples	Illumina	20	[20]	0	11
Human papillomavirus community in healthy persons	Illumina	20^a^	[21]	9	5
Identification of hepatotropic viruses from plasma	Illumina	14	[18]	1	4
Intestinal bacterial and RNA viral communities from sentinel birds	Ion Torrent	8	[24]	7	0
Intestinal virome in healthy and diarrhoeic neonatal piglets	Ion Torrent	29	[27]	3	2
Metagenomic analysis for severe acute respiratory infection	Illumina	4	[29]	0	4
Metagenomic identification of viral pathogens in clinical samples	Illumina	3	[25]	1	1
New viral sequences identified in Asian citrus psyllid	Illumina	4	[26]	1	3
Novel adenovirus associated with baboon acute respiratory outbreak	Illumina	4	[17]	3	1
Novel human pegivirus associated with hepatitis C virus co-infection	Illumina	15	[23]	11	2
RNA sequencing in influenza virus-positive respiratory samples	Illumina	49	[9]	16	13
RNA-seq of RNA viruses from faecal and blood samples	Illumina	82	[8]	6	76
Simultaneous sequencing of multiple RNA virus genomes	Ion Torrent	40	[22]	33	4
Viral genome-targeted assembly pipeline	Illumina	1	[13]	1	0
Virus identification in unknown tropical febrile illness cases	Illumina	1^b^	[16]	1	0

^a^Selected runs with assembled contigs >1000 bp [21].

^b^A selected run with mapped viral reads >5000 [16].

## Results

### drVM pipeline

**Figure 2.**
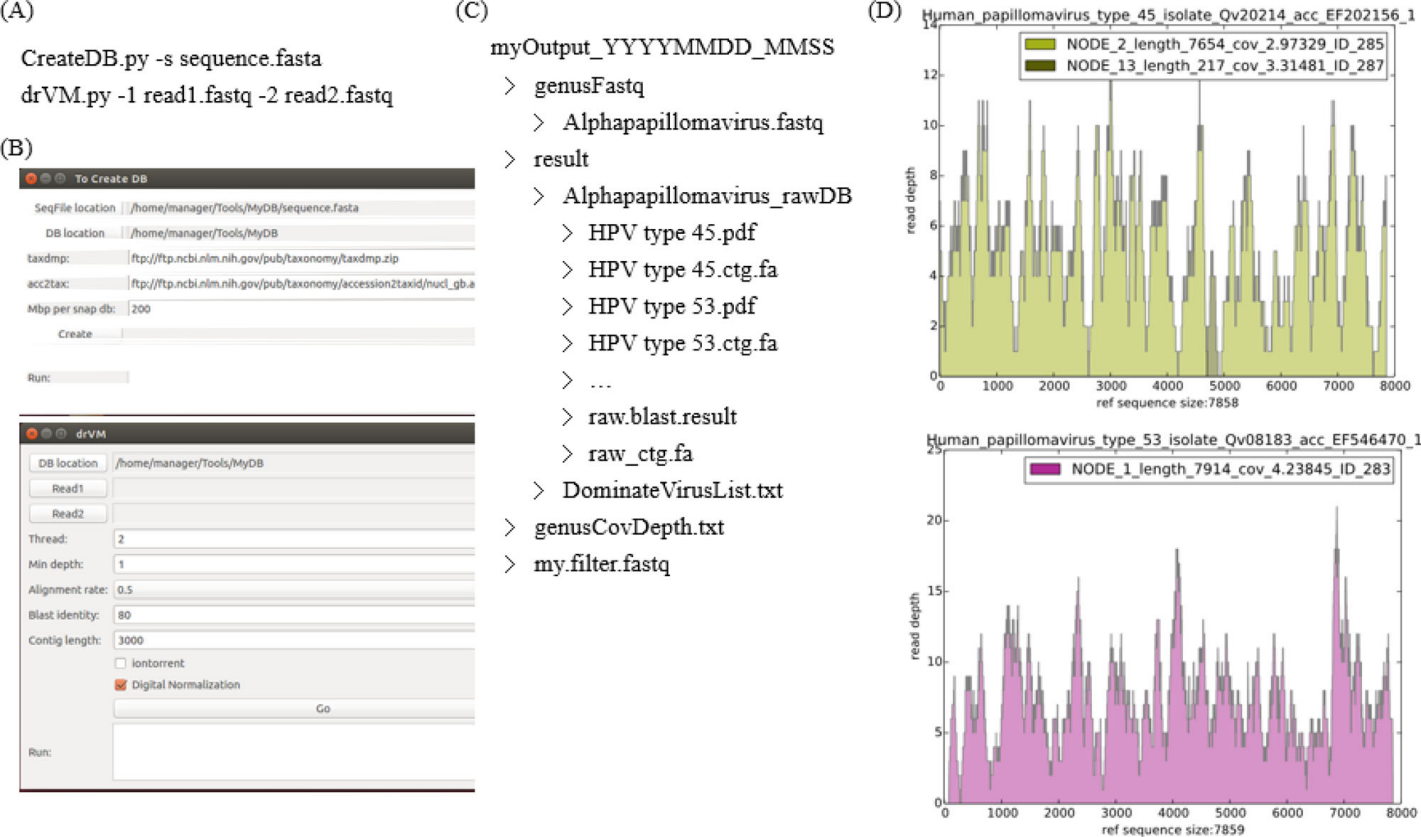
Input and output explanation of drVM. (A) Input associated with the command-line interface. (B) Input associated with the graphical user interface. (C) Output file structure generated by drVM. (D) Coverage profiles produced by drVM.

drVM was designed to detect and reconstruct known viral genomes from metagenomes. As described in the Materials and Methods, it contains two modules: CreateDB.py and drVM.py (Fig. 1). The viral sequences downloaded from NCBI were processed by CreateDB.py to produce SNAP and BLAST databases. Taxonomy information was also automatically extracted from NCBI. In drVM.py, metagenomic reads that were aligned to complete viral sequences were firstly partitioned into genus-level groups to facilitate *de novo* assembly of classified reads. Each genus-level assembly was then annotated with a close reference in refDB or rawDB to produce corresponding coverage plots. The drVM pipeline was implemented, in Python, to incorporate open-source tools including BLAST, khmer, SNAP, SOAP2, and SPAdes. The software is open-source and available for download: https://sourceforge.net/projects/sb2nhri/files/drVM/. In addition to the drVM script, the module is distributed as a Docker image in the Docker Hub (https://hub.docker.com/r/990210oliver/drvm/) repository, an Amazon Machine Image (AMI) in Community AMIs, and as a virtual machine image (drVM.ova) on the sourceforge website. The instructions for users can be found in Supplementary file 2. With the Docker, Amazon machine image, and the drVM virtual machine, users are able to interface with drVM with ease. In addition to executing commands in a terminal window (Fig. 2A), a graphical user interface (Fig. 2B) was created in the drVM virtual machine. Users are thus able to construct the required databases for drVM by simply clicking on the ‘Create’ button featured in CreateDB.py. Following a 30-minute period during which DB is created, users are able to analyze metagenomic data with drVM.py to produce a folder (Fig. 2C) containing assembled viral genomes (*.ctg.fa) along with annotated coverage plots (Fig. 2D). Note that viral sequences were fully assembled, *de novo*, by SPAdes using reads within the same genus, and each assembled contig (e.g. NODE_2_length_7654 and NODE_1_length_7914 in Fig. 2D) was annotated with its closely related genome (e.g. Human papillomavirus type 45 and type 53). The coverage plots were generated by plotting read coverage across genomic position, with the y-axis representing read depth.

### Viral genome reconstruction using simulated data

**Figure 3.**
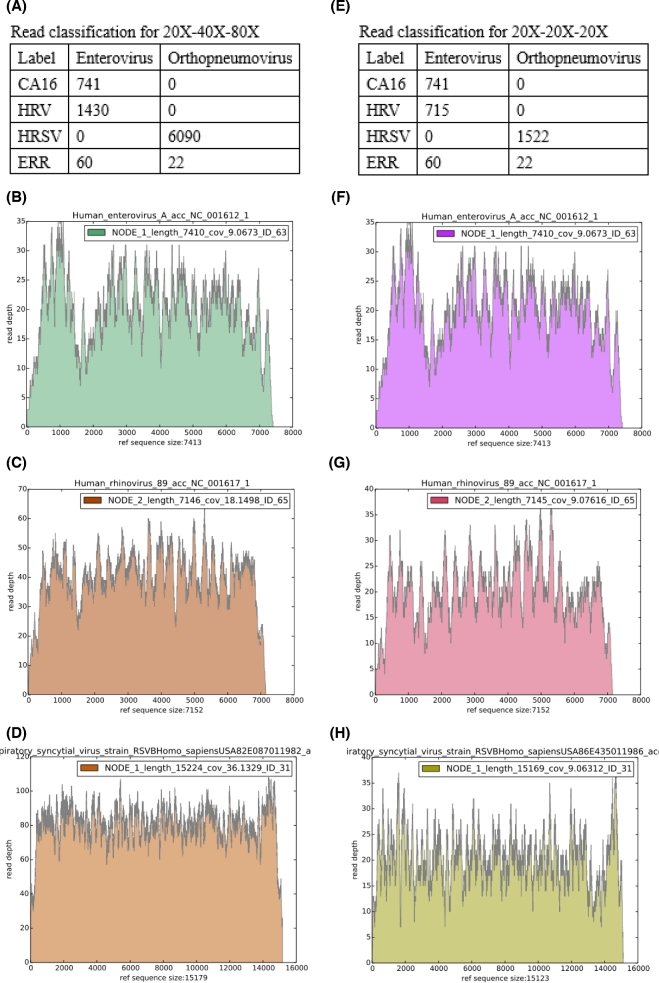
drVM results for read mixture simulations of human enterovirus (CA16), human rhinovirus (HRV), human respiratory syncytial virus (HRSV), and reads from ERR690488. (A-D) 20X-40X-80X: 741 CA16, 1430 HRV, and 6090 HRSV reads. (E-H) 20X-20X-20X: 741 CA16, 715 HRV, and 1522 HRSV reads.

To reconstruct the 9646-bp HCV genome from the simulated datasets (485–2425 100-bp paired-end HCV reads for 10× to 50× HCV + 3.5 million SRR3031107 liver cancer cell paired-end reads + 1.6 million SRR062412 HMP paired-end reads), drVM first placed all the HCV (485–2425) along with 105 liver and 274 HMP paired-end reads into fastq files classified under the genus “Hepacivirus.” It proceeded to assemble the reads into a HCV genome (length ranging from 9631 to 9644 bp) with 100% sequence identity to the reference genome (NC_004102.1) as the depth of HCV reads was ≧20×. Hence, based on this simulation study, the minimum read depth for drVM to reconstruct the viral genome was 20×. To assess the capability of assembling mixed viral genomes, two simulation datasets have been used: 20×-40×-80× and 20×-20×-20× for a mixture of reads of CA16, HRV, and HRSV combined with 1-Gbp reads from an influenza-negative respiratory sample (ERR690488). The results generated by drVM for 20×-40×-80× and 20×-20×-20× are shown in Fig. 3A-D and E-H, respectively. drVM correctly classified viral reads into the corresponding genus fastq files (CA16 and HRV reads in Enterovirus and HRSV in Orthopneumovirus, shown in Fig. 3A and E) and successfully reconstructed the three viral genomes (Fig. 3B-D and F-H) in the two genus folders. The assembled sequences of length 7145 to 15 224 bp were 100% identical to their reference genomes. The simulations revealed that few bacterial and host reads (<0.02%, 274 of 1.6 million) were classified into the viral genus-level fastq files, and the existence of background reads did not interfere with viral assembly.

### Detection and reconstruction of viral genomes

Three hundred and forty-nine sequencing datasets from 18 research studies (Table 1 and Table S1) were downloaded and analyzed by means of drVM. The results generated by the drVM package (except filtered reads) for the 349 runs are provided at http://sb.nhri.org.tw/drVM/, and the assembled viral sequences (also in Table S1), along with their corresponding coverage plots, are available for download. As shown in Table S1 and the website, drVM produced coverage plots in analyzing metagenomics reads of 260 sequencing runs and reconstructed viral genomes from 147 runs by presenting a single contig mapped on a reference. Here, we take the second study listed in Table 1 as an example; the authors described sequence-independent amplification for samples containing ultra-low amounts of viral RNA coupled with 34-run Illumina sequencing (SRR513075 to SRR527726 and SRR629705-9 listed in Table S1). *De novo* assembly, optimized for viral genomes, was performed so as to capture 96% to 100% of the viral protein coding region of the human immunodeficiency virus (HIV), respiratory syncytial virus (RSV), and West Nile virus (WNV); 31 consensus genome assemblies have been submitted to NCBI (JX503071-JX503101) [19]. These viruses were successfully detected by drVM in separate samples through the creation of coverage plots for the 34 sequencing runs (see http://sb.nhri.org.tw/drVM/). drVM reconstructed complete viral genomes in 25 runs, thus the remaining 9 runs are labeled as “detection” in Table 1. The assembled genome sequences such as HIV in SRR513075, WNV in SRR527701, and RSV in SRR527708 can be found in Table S1. Although drVM did not reconstruct HIV genomes for JX503077 and JX503083 from SRR513078 and SRR527710, respectively, it produced HIV contigs with length >4000 bp. The drVM-produced viral sequences shared over 99.9% average sequence identity to the reference genomes (20 pairs of reference and assembled sequence). In addition to HIV, RSV, and WNV, drVM reconstructed GB virus C genomes in SRR513075, SRR513080, SRR513087, SRR629705, and SRR629708. The maximum identity of the assembled sequences to the reference genomes deposited in NCBI's Nucleotide collection (nr/nt) is 93%, which reveals that these assembled sequences have yet to be published. drVM also detected hepatitis C viruses in SRR513080 (three assembled HCV contigs > 1 kbp), SRR629705 (one HCV contig > 5 kbp), and SRR629708; torque teno virus (contigs > 1.5 kbp) was also detected in SRR527718, SRR527725, SRR629705, and SRR629707. This finding has, to date, not been reported in the literature. Furthermore, 12 complete or near-complete viral genomes of viruses including adenovirus, norovirus, and hepatitis B virus have been obtained and submitted to NCBI (KJ194499-KJ194510) in the fourth study [20]; drVM automatically produced nine complete genomes among them, with over 99.9% average sequence identity to the reference genomes (Table S1). In analyzing reads of the 20 sequencing runs, drVM reconstructed 17 viral genomes in 11 runs and did not produce any coverage plot for the other 9 runs (Table 1). An additional eight viral genomes, belonging to plant viruses (including cucumber green mottle mosaic virus, paprika mild mottle virus, pepper mild mottle virus, tobacco mosaic virus, and tomato mosaic virus), were assembled by drVM. Genomes of the four plant viruses, with the exception of pepper mild mottle virus, have undergone complete assembly by the prior study [20], albeit the plant viral sequences provided in Table S1 have not been published. As for the fourth study from the bottom of Table 1, the RNA shotgun sequencing method has been used in 82 samples, but only 17 sequences of norovirus (HF952119-HF952135) have been submitted to NCBI [8]. drVM produced coverage plots for all 82 sequencing runs (shown on the website) and assembled two hepatitis C virus genomes, 74 norovirus genomes, two human picobirnavirus genomes, and one pepino mosaic virus genome from 76 sequencing runs (Table S1). Over 99.98% average sequence identity was obtained when the drVM-produced sequences were compared to the 17 corresponding references. To illustrate the wide-ranging versatility of drVM, it was also leveraged to detect/reconstruct viral genomes from respiratory samples [9], the fifth study from the bottom of Table 1. drVM produced 2 complete influenza A H1N1 virus genomes (ERR690494 and ERR690532 in http://sb.nhri.org.tw/drVM/) and 10 H3N2 virus genomes (e.g., ERR690510) in 12 influenza-positive samples and one human respiratory syncytial virus in a negative control sample (ERR690491). Taken together, various viral genomes including DNA, RNA, and retro-transcribing viruses (Table 2) have been reconstructed by drVM; this has been applied to the analysis of 349 metagenomic datasets from 18 studies. For example, drVM assembled a human adenovirus D genome with length of 34 901 bp in ERR233414, a human bocavirus (ssDNA) genome with length of 5250 bp, and a genome of human parainfluenza virus (ssRNA negative strand) with length of 15519 bp in SRR2010686, two segments of human picobirnavirus (dsRNA) with lengths of 2485 and 1717 bp in ERR227885, a bovine viral diarrhea virus (ssRNA positive strand) with length of 12 216 bp in SRR1170806, and a hepatitis B virus (retrovirus) genome with length of 3166 bp in ERR233420. In summary, drVM successfully reconstructed over 200 viral genomes of 30 viral species (Table 2) among 349 metagenomic sequencing runs. Over 150 assembled viral genomes are provided in Table S1.

**Table 2.** Various viral genomes reconstructed by drVM based on the corresponding NGS reads

Virus type	Viral genome reconstruction by drVM
dsDNA	Human adenovirus (ERR233414), Human papillomavirus (ERR233428), Simian adenovirus (SRR766764)
ssDNA	Adeno-associated virus (SRR766763), Bovine parvovirus (SRR2010686), Fox circovirus (SRR1920168), Human bocavirus (SRR2010686), Torque teno mini virus (SRR2040553), Torque teno virus (SRR2010686)
dsRNA	Human picobirnavirus (ERR227885)
ssRNA (+)	Bovine viral diarrhea virus (SRR1170806), Chikungunya virus (SRR3180716), GB virus C (SRR544883), Hepatitis C virus (SRR2940645), Human pegivirus (SRR2940645), Human rhinovirus (SRR2010685), Norovirus (ERR233428), Pepino mosaic virus (ERR227848), Pepper mild mottle virus (ERR233431), Porcine kobuvirus (ERR1097471), Tobacco mild green mosaic virus (ERR233421), Tobacco mosaic virus (ERR233421), Tomato mosaic virus (ERR233424), West Nile virus (SRR527705)
ssRNA (−)	Human parainfluenza virus (SRR2010686), Human respiratory syncytial virus (ERR690491), Respiratory syncytial virus (SRR527708), Influenza A virus (ERR690510)
Retro-transcribing	Hepatitis B virus (ERR233420), Human immunodeficiency virus (SRR513075)

### Performance comparison

Unlike SURPI [10], VIP [12], and VirusTAP [13], drVM directly identifies reads by aligning them to complete viral sequences and therefore is expected to save a substantial amount of computational time from subtracting reads of the host and co-located bacteria. Three datasets – SRR1170797, SRR1106548, and DRR049387 – used in VIP, SURPI, and VirusTAP were separately input into them for the pipeline evaluation [10, 12, 13]. Two sequencing runs comprising co-existing multiple human papillomavirus types [21] and segmented-genome influenza viruses [9] were used as additional datasets for performance comparison. As demonstrated in Table 3, drVM requires the shortest execution time with regards to SRR1106548 and DRR049387 and the second-shortest time for the other three datasets. Nevertheless, it produced a complete genome of bovine viral diarrhea virus (genome size: 12.5 kbp) in SRR1170797, two complete genomes of human papillomavirus type 45 and type 53 (genome size: ∼7.8 kbp) in SRR062073, and eight complete segments of influenza A virus H3N2 in ERR690519. Commands and results can be found in Supplementary information. Note that the analyses of drVM, SURPI, and VIP (in Table 3) were performed on a quad-core CPU with 128 GB RAM while VirusTAP was executed on a 120-core CPU web server with 1 TB RAM. Therefore, drVM was validated to be the most rapid tool for virus identification when computational hardware was considered. Moreover, drVM reconstructed the complete viral genomes from metagenomics reads of SRR1170797, SRR062073, and ERR690519 runs, while the other three tools were not able to produce complete genomes for these viruses. The outputs generated by each tool running on the datasets delineated in Table 3 are available at: https://sourceforge.net/projects/sb2nhri/files/drVM/Comparison/. Please also note that the results are reproducible even when running a drVM virtual machine with a configured memory size of 8 GB on a Windows system with Intel Xeon E31248 CPU and 32 GB RAM (see Supplementary information). To simulate the disturbance of host reads, *homo sapiens* raw reads from liver cancer cells (SRR3031107) were concatenated to sequencing reads (SRR544883) from the hepatitis C virus infection [18]. Before read concatenation, drVM reconstructed a GB virus C genome (length of 9244 bp) from 101 364 paired-end reads in fastq files of Pegivirus in SRR544883, as shown in Table 2. Although the software managed to reconstruct the viral genome of GB virus C from the concatenated reads, execution time increased by a factor of three (from 43 minutes to 2 hours 7 minutes while analyzing read bases of 711 Mb to 10 Gb). Please note that SRR3031107 reads were not classified into fastq files of Pegivirus when the concatenated reads (SRR3031107 + SRR544883) were employed as inputs to drVM. It was therefore confirmed that host read subtraction is not necessary in drVM. Taken together, drVM not only facilitates the rapid detection of viral reads but also enables the *de novo* assembly of the classified reads into viral genomes.

**Table 3.** Performance comparison between drVM, SURPI, VIP, and VirusTAP

Target virus (run accession)	Read bases (Mbp)		drVM	SURPI (comprehensive)	VIP (sense)	VirusTAP
Bovine viral diarrhea virus (SRR1170797)	12.5	Run time^a^	149 sec	52** **365 sec	1683 sec	71 sec
		Result^b^	**12 224 bp**	262 bp	9078 bp	353 bp
Human immunodeficiency virus (SRR1106548)^c^	600.9	Run time	598 sec	32** **604 sec	25** **049 sec	1388 sec
		Result	3055 bp	799 bp	4632 bp	2896 bp
Human papillomavirus (SRR062073)	5000	Run time	608 sec	18** **107 sec	8603 sec	519 sec
		Result	**7654 & 7914 bp**	3515 bp	2164 bp	555 bp
Human rotavirus A (DRR049387)	266.1	Run time	464 sec	59** **510 sec	6259 sec	925 sec
		Result	13 contigs	13 contigs	41** **377 reads	**11 viral contigs**
Influenza A virus (ERR690519)	3300	Run time	12** **697sec	16** **997 sec	86** **001 sec	4504 sec
		Result	**8 H3N2 segments**	11 contigs	2673 reads	34 viral contigs

^a^The analyses were executed on a quad-core CPU with 128 GB RAM, except for VirusTAP (executed on a 120-core CPU with 1 TB RAM). Please note that drVM is able to run these datasets with 8 GB RAM (Supplementary information).

^b^The result is summarized as the largest length of viral contig (bold: close to the genome size of target virus), read number mapped to the target virus, or contig number depending on the output of tools and the target virus (Human rotavirus A: 11 segments, Influenza A virus: 8 segments).

^c^The results were produced by analyzing reads with barcode: GCCAAT, except for SURPI, which recognizes multiple barcodes.

## Discussion

As NGS technology is becoming a more common means to detect pathogens in clinical samples, our goal is to establish a simple and effective pipeline that allows accurate and rapid viral genome reconstruction from metagenomic NGS data generated from complex clinical samples. As described in SURPI, SNAP executes 10–100 × more rapidly than existing alignment tools including Bowtie 2 [37] and BWA [38]. drVM and SURPI use SNAP while VIP and VirusTAP utilize Bowtie 2 and BWA-SW [39], respectively. SNAP coupling with the light reference database (viral sequences vs. human, bacterial, and viral sequences used in SURPI, VIP, and VirusTAP) yields a noteworthy reduction in the processing time for viral read identification in drVM. Furthermore, the reference database can easily be updated whenever necessary (see Supplementary information). In contrast, SURPI classifies reads against viral and bacterial databases to identify all potential pathogens, which invariably requires extended execution time and requires more effort with regards to ensuring that the references are updated and current. In drVM, viral reads with the loosest edit distance of 24 (in SNAP) were partitioned into genus-level groups based on the virus taxonomy retrieved from the NCBI Taxonomy databases, excluding phages. The filtered reads belonging to a viral genus are therefore allowed to be divergent from reference genomes so long as there is one contiguous seed of 20 bases matching exactly to a reference. However, since drVM relies on known viral sequences for viral read identification and genus-level partition, it may not be able to discover novel viruses that are very different to known viruses. For subsequent *de novo* assembly, digital normalization was then employed to correct uneven read depth distribution so as to eliminate redundant reads while retaining sufficient information. For example, drVM produced a 7455-bp contig of porcine kobuvirus for ERR1097471 (Table 2, the assembled sequence in Table S1) but produced a fragmented assembly when digital normalization was not used (-dn off). This assembled sequence shared only 90% identity at nucleotide level with a closely related porcine kobuvirus genome sequence deposited in NCBI's Nucleotide collection (nr/nt), hence demonstrating that drVM is able to produce a complete genome sequence of porcine kobuvirus from the intestinal virome in neonatal piglets [27]. Additionally, normalization enables us to reduce the processing time required for assembly. For example, not activating the digital normalization routine in drVM increased the execution time of ERR1097472 from 20 minutes to 3 hours. It is important to note that the digital normalization routine is employed to down-sample reads, insinuating that the results produced by drVM can vary (e.g., SRR527703 in http://sb.nhri.org.tw/drVM/) from run to run. The normalized reads were assembled, in a *de novo* fashion, by SPAdes with multiple k-mer sizes (21, 33, 55… automatically selected based on read length). Since SPAdes operates with Illumina and Ion Torrent reads, drVM is able to handle sequencing reads produced by these two platforms (default for Illumina, -type iontorrent for Ion Torrent). Moreover, drVM annotates each genus-level assembly with a close reference to produce the corresponding coverage plots. If multiple contigs are present in one coverage plot, reads aligned to the contigs are extracted in pairs for subsequent re-assembly; such a process is able to improve genome completeness. An example can be seen in the run corresponding to SRR527705 – fragmented contigs were annotated with the WNV in the pre.run routine; drVM produced a 11 017-bp contig of the complete genome (see http://sb.nhri.org.tw/drVM/ for the details). Over 99.98% of the target sequence identity was obtained in the drVM-produced contig when referenced against the submitted assembly (JX503096.1) [19], as shown in Table S1.

In the comparative study of VIP and VirusTAP, the authors have compared their assemblies with direct metagenomic assemblies via Ensemble Assembler, IDBA_UD, A5-miseq, and CLC workbench. The results have led the authors to conclude that “classification to assembly” outperformed direct assembly in terms of assembly continuity and execution time [12, 13], suggesting that to extract viral reads from metagenomes is a prior step to sequence assembly. Although drVM was not compared with SPAdes, it was compared against SURPI, VIP, and VirusTAP. As evident from Table 3, drVM produced complete genomes of bovine viral diarrhea virus and human papillomavirus in the analysis of reads produced by Ion Torrent and Illumina platforms, respectively; the other tools were not able to assemble complete genomes for these viruses. Although drVM produced distinct viral genome assemblies within the same genus, for human papillomavirus types 45 and 53 in SRR062073 (Fig. 2 and Table 3) and for human enterovirus rhinovirus in the simulation dataset (Fig. 3), the assembler may not be able to handle mixtures of very closely related viruses. Moreover, a biased viral reference database may result in assemblies with missing segments or no assembly whatsoever. Unlike SURPI and VIP, the coverage plots produced by drVM are created by mapping raw reads back to the assembled contigs, not to closely-related references. A coverage plot with a continuous profile reflects the accuracy and continuity of the assembly paradigm. With the support of coverage plot, one can rest assured that viruses with drVM-produced genome assemblies are present in the sample. Among SURPI, VIP, and VirusTAP, VirusTAP is the only user-friendly tool for viral genome assembly from metagenomic reads. The other two pipelines are not easily operated by users lacking advanced bioinformatics skills. Although academic users can access VirusTAP and obtain viral genomes with ease, the package is not suitable for assembling Ion Torrent data (Table 3, SRR1170797) and low-copy viruses (Table 3, SRR062073). Moreover, users are not able to update reference databases in VirusTAP, a web-based tool. Alternatively, drVM provides various images for Docker, Amazon, and Virtual Machine deployment and features a user-friendly interface (as shown in Fig. 2B) in the Virtual Machine. drVM also generates self-explanatory results (Fig. 2C and D) accurately and rapidly; it therefore is well-suited for clinical applications.

Although drVM operates in such a manner that the subtraction of the host read is neglected, which may produce valid host sequences annotated with viral references, this error can be easily identified via inspection of the assembled contigs. For example, analyzing SRR3031107 concatenated with SRR544883, a 1872-bp contig annotated with Feline leukemia virus gene for viral Notch2 (92% identity) actually corresponded to *homo sapiens* notch 2 (100% identity). Nevertheless, the provirus that integrated into a host genome is partial and it can be easily distinguished from the complete viral genome (Feline leukemia virus, genome size: 8.45 kbp) by length. We therefore recommend that conclusions drawn from drVM should be made with caution, especially with regards to retrovirus detection. We have applied drVM in the analyses of over 300 sequencing runs retrieved from NCBI's SRA to demonstrate that drVM is indeed able to efficiently produce genome assemblies for known eukaryotic viruses from metagenomic data.

## Availability and requirements

Project name: drVM

Project home page: https://sourceforge.net/projects/sb2nhri/files/drVM/

Operating system(s): OS X, Linux, Windows

Programming language: Python

Requirements: Amazon machine image, Docker, or Virtual machine; 8 GB RAM

License: GNU General Public License, version 3.0 (GPL-3.0)

## Availability of supporting data

Snapshots of the code and further supporting data is available in the GigaScience repository GigaDB [40].

## Abbreviations

AMI: Amazon machine image; HCV: hepatitis C virus; HIV: immunodeficiency virus; NGS: next-generation sequencing; RSV: respiratory syncytial virus; SRA: sequencing read archive; WNV: West Nile virus.

## Supplementary data

Supplementary data are available at *GIGSCI* online.

**Supplementary Table S1:** list of 349 sequencing datasets from 18 research studies.

**Supplementary File 2:** supplementary information.

GIGA-D-16-00060_Original_Submission.pdf

GIGA-D-16-00060_Revision_1.pdf

GIGA-D-16-00060_Revision_2.pdf

Responses_to_reviewer_comments_Orginal_Submission.pdf

Response_to_Reviewer_Comments_Revision_1.pdf

Reviewer_1_Report_(Revision_1).pdf

Reviewer_1_Report_Original_Submission.pdf

Reviewer_2_Report_(Revision_1).pdf

Reviewer_2_Report_Original_Submission.pdf

Reviewer_3_Report_(Revision_1).pdf

Reviewer_3_Report_Original_Submission.pdf

Supplemental material **Supplementary Table S1:** list of 349 sequencing datasets from 18 research studies.
**Supplementary File 2:** supplementary information.

## Competing interests

The authors declare that they have no competing interests.

## Author contributions

YCL conceived the project. HHL implemented the pipeline. YCL and HHL prepared, read, and approved the final manuscript.

## References

[1] Brister JR, Ako-Adjei D, Bao Y *et al* NCBI viral genomes resource. Nucleic Acids Res 2015;43:D571–7. 25428358 10.1093/nar/gku1207 PMC4383986

[2] Pickett BE, Sadat EL, Zhang Y *et al* ViPR: an open bioinformatics database and analysis resource for virology research. Nucleic Acids Res 2012;40:D593–8. 22006842 10.1093/nar/gkr859 PMC3245011

[3] Sharma D, Priyadarshini P, Vrati S Unraveling the web of viroinformatics: computational tools and databases in virus research. J Virol 2015;89:1489–501. 25428870 10.1128/JVI.02027-14 PMC4300767

[4] Chan PK. Outbreak of avian influenza A(H5N1) virus infection in Hong Kong in 1997. Clin Infect Dis 2002;34(Suppl 2):S58–64. 11938498 10.1086/338820

[5] Bean AG, Baker ML, Stewart CR *et al* Studying immunity to zoonotic diseases in the natural host - keeping it real. Nat Rev Immunol 2013;13:851–61. 24157573 10.1038/nri3551 PMC7098194

[6] Feldmann H. Ebola–a growing threat? N Engl J Med 2014;371:1375–8. 24805988 10.1056/NEJMp1405314

[7] Calvet G, Aguiar RS, Melo ASO *et al* Detection and sequencing of Zika virus from amniotic fluid of fetuses with microcephaly in Brazil: a case study. The Lancet Infect Dis 2016;16:653–60. 10.1016/S1473-3099(16)00095-5 26897108

[8] Batty EM, Wong TH, Trebes A *et al* A modified RNA-Seq approach for whole genome sequencing of RNA viruses from faecal and blood samples. PLoS One 2013;8:e66129. 23762474 10.1371/journal.pone.0066129 PMC3677912

[9] Fischer N, Indenbirken D, Meyer T *et al* Evaluation of unbiased next-generation sequencing of RNA (RNA-seq) as a diagnostic method in influenza virus-positive respiratory samples. J Clin Microbiol 2015;53:2238–50. 25972420 10.1128/JCM.02495-14 PMC4473199

[10] Naccache SN, Federman S, Veeraraghavan N *et al* A cloud-compatible bioinformatics pipeline for ultrarapid pathogen identification from next-generation sequencing of clinical samples. Genome Res 2014;24:1180–92. 24899342 10.1101/gr.171934.113 PMC4079973

[11] Flygare S, Simmon K, Miller C *et al* Taxonomer: an interactive metagenomics analysis portal for universal pathogen detection and host mRNA expression profiling. Genome Biol 2016;17:111. 27224977 10.1186/s13059-016-0969-1 PMC4880956

[12] Li Y, Wang H, Nie K *et al* VIP: an integrated pipeline for metagenomics of virus identification and discovery. Sci Rep 2016;6:23774. 27026381 10.1038/srep23774 PMC4824449

[13] Yamashita A, Sekizuka T, Kuroda M VirusTAP: Viral genome-targeted assembly pipeline. Front Microbiol 2016;7:32. 26870004 10.3389/fmicb.2016.00032 PMC4735447

[14] Merkel D Docker: lightweight Linux containers for consistent development and deployment. Linux J 2014;2014:2.

[15] Nocq J, Celton M, Gendron P *et al* Harnessing virtual machines to simplify next-generation DNA sequencing analysis. Bioinformatics 2013;29:2075–83. 23786767 10.1093/bioinformatics/btt352

[16] Yozwiak NL, Skewes-Cox P, Stenglein MD *et al* Virus identification in unknown tropical febrile illness cases using deep sequencing. PLoS Negl Trop Dis 2012;6:e1485. 22347512 10.1371/journal.pntd.0001485 PMC3274504

[17] Chiu CY, Yagi S, Lu X *et al* A novel adenovirus species associated with an acute respiratory outbreak in a baboon colony and evidence of coincident human infection. MBio 2013;4:e00084. 23592261 10.1128/mBio.00084-13 PMC3634605

[18] Law J, Jovel J, Patterson J *et al* Identification of hepatotropic viruses from plasma using deep sequencing: a next generation diagnostic tool. PLoS One 2013;8:e60595. 23613733 10.1371/journal.pone.0060595 PMC3629200

[19] Malboeuf CM, Yang X, Charlebois P *et al* Complete viral RNA genome sequencing of ultra-low copy samples by sequence-independent amplification. Nucleic Acids Res 2013;41:e13. 22962364 10.1093/nar/gks794 PMC3592391

[20] Cotten M, Oude Munnink B, Canuti M *et al* Full genome virus detection in fecal samples using sensitive nucleic acid preparation, deep sequencing, and a novel iterative sequence classification algorithm. PLoS One 2014;9:e93269. 24695106 10.1371/journal.pone.0093269 PMC3973683

[21] Ma Y, Madupu R, Karaoz U *et al* Human papillomavirus community in healthy persons, defined by metagenomics analysis of human microbiome project shotgun sequencing data sets. J Virol 2014;88:4786–97. 24522917 10.1128/JVI.00093-14 PMC3993818

[22] Neill JD, Bayles DO, Ridpath JF Simultaneous rapid sequencing of multiple RNA virus genomes. J Virol Methods 2014;201:68–72. 24589514 10.1016/j.jviromet.2014.02.016 PMC7119728

[23] Berg MG, Lee D, Coller K *et al* Discovery of a novel human pegivirus in blood associated with hepatitis C virus co-infection. PLoS Pathog 2015;11:e1005325. 26658760 10.1371/journal.ppat.1005325 PMC4676677

[24] Day JM, Oakley BB, Seal BS *et al* Comparative analysis of the intestinal bacterial and RNA viral communities from sentinel birds placed on selected broiler chicken farms. PLoS One 2015;10:e0117210. 25635690 10.1371/journal.pone.0117210 PMC4311960

[25] Greninger AL, Naccache SN, Federman S *et al* Rapid metagenomic identification of viral pathogens in clinical samples by real-time nanopore sequencing analysis. Genome Medicine 2015;7. 10.1186/s13073-015-0220-9 PMC4587849 26416663

[26] Nouri S, Salem N, Nigg JC *et al* Diverse array of new viral sequences identified in worldwide populations of the Asian citrus psyllid (Diaphorina citri) using viral metagenomics. J Virol 2015;90:2434–45. 26676774 10.1128/JVI.02793-15 PMC4810699

[27] Karlsson OE, Larsson J, Hayer J *et al* The intestinal eukaryotic virome in healthy and diarrhoeic neonatal piglets. PLoS One 2016;11:e0151481. 26982708 10.1371/journal.pone.0151481 PMC4794121

[28] Lojkic I, Bidin M, Prpic J *et al* Faecal virome of red foxes from peri-urban areas. Comp Immunol Microbiol Infect Dis 2016;45:10–15. 27012914 10.1016/j.cimid.2016.01.005 PMC7112549

[29] Wang Y, Zhu N, Li Y *et al* Metagenomic analysis of viral genetic diversity in respiratory samples from children with severe acute respiratory infection in China. Clin Microbiol Infect 2016;22:458. 10.1016/j.cmi.2016.01.006 PMC7172101 26802214

[30] Zaharia M, Bolosky WJ, Curtis K *et al* Faster and more accurate sequence alignment with SNAP. arXiv preprint arXiv:11115572 2011.

[31] Camacho C, Coulouris G, Avagyan V *et al* BLAST+: architecture and applications. BMC Bioinformatics 2009;10:421. 20003500 10.1186/1471-2105-10-421 PMC2803857

[32] Bankevich A, Nurk S, Antipov D *et al* SPAdes: a new genome assembly algorithm and its applications to single-cell sequencing. J Comput Biol 2012;19:455–77. 22506599 10.1089/cmb.2012.0021 PMC3342519

[33] Howe AC, Jansson JK, Malfatti SA *et al* Tackling soil diversity with the assembly of large, complex metagenomes. Proc Natl Acad Sci U S A 2014;111:4904–9. 24632729 10.1073/pnas.1402564111 PMC3977251

[34] Crusoe MR, Alameldin HF, Awad S *et al* The khmer software package: enabling efficient nucleotide sequence analysis. F1000Res 2015;4:900. 26535114 10.12688/f1000research.6924.1 PMC4608353

[35] Li R, Yu C, Li Y *et al* SOAP2: an improved ultrafast tool for short read alignment. Bioinformatics 2009;25:1966–7. 19497933 10.1093/bioinformatics/btp336

[36] Boisvert S, Raymond F, Godzaridis E *et al* Ray meta: scalable de novo metagenome assembly and profiling. Genome Biol 2012;13:R122. 23259615 10.1186/gb-2012-13-12-r122 PMC4056372

[37] Langmead B, Salzberg SL Fast gapped-read alignment with Bowtie 2. Nat Methods 2012;9:357–9. 22388286 10.1038/nmeth.1923 PMC3322381

[38] Li H, Durbin R Fast and accurate short read alignment with Burrows-Wheeler transform. Bioinformatics 2009;25:1754–60. 19451168 10.1093/bioinformatics/btp324 PMC2705234

[39] Li H, Durbin R Fast and accurate long-read alignment with Burrows-Wheeler transform. Bioinformatics 2010;26:589–595. 20080505 10.1093/bioinformatics/btp698 PMC2828108

[40] Lin H, Liao Y Supporting data for “drVM: a new tool for efficient genome assembly of known eukaryotic viruses from metagenomes” GigaScience Database. 2017 http://dx.doi.org/10.5524/100272. 10.1093/gigascience/gix003 PMC5466706 28369462

